# Supplementary material for: Influence of Sports Activities on Prosocial Behavior of Children and Adolescents: A Systematic Literature Review
Source: Int J Environ Res Public Health. 2022 May 26;19(11):6484. doi: 10.3390/ijerph19116484 (PMC9180162; doi:10.3390/ijerph19116484)
Supplement: Supplementary file 1 [file ijerph-19-06484-s001.zip › ijerph-1700872-supplementary.pdf]

Table S1. Search strategies.

Pub med 3364<br>((sports movement[Title] OR physical exercise[Title] OR sports activities[Title] OR sport*[Title] OR motor[Title] OR athletic sports[Title]) AND (child[Title] OR children[Title] OR childhood[Title] OR pediatric[Title] OR infant[Title] OR kids[Title] OR adolescent*[Title] OR teens[Title] OR teenager*[Title] OR juvenile[Title] OR school-aged children[Title])) AND (altruism[Title/Abstract] OR humanitarianism[Title/Abstract] OR prosocial behavior[Title/Abstract] OR social behavior[Title/Abstract] OR compassion[Title/Abstract] OR help*[Title/Abstract] OR care[Title/Abstract] OR caring[Title/Abstract] OR empath*[Title/Abstract] OR shar*[Title/Abstract] OR donat*[Title/Abstract] OR comfort*[Title/Abstract] OR OR[Title/Abstract])
Web of science 2251<br>((TI=(sports movement OR physical exercise OR sports activities or sport* OR motor or athletic sports)) AND TI=(child OR children OR childhood OR pediatric OR infant OR kids OR adolescent* OR teens OR teenager* OR juvenile OR school-aged children)) AND TS=(altruism OR humanitarianism OR prosocial behavior OR prosocial behavior OR social behavior OR compassion OR help* OR care OR caring OR empath* OR shar* OR donat* OR comfort*)
Eric 1764<br>AB (sports movement OR physical exercise OR sports activities or sport* OR motor or athletic sports) AND AB (child OR children OR childhood OR pediatric OR infant OR kids OR adolescent* OR teens OR teenager* OR juvenile OR school-aged children) AND AB (altruism OR humanitarianism OR prosocial behavior OR prosocial behavior OR social behavior OR compassion OR help* OR care OR caring OR empath* OR shar* OR donat* OR comfort*)
Psychology and Behavioral Sciences Collection 995<br>SU (sports movement OR physical exercise OR sports activities or sport* OR motor or athletic sports) AND SU (child OR children OR childhood OR pediatric OR infant OR kids OR adolescent* OR teens OR teenager* OR juvenile OR school-aged children) AND TX (altruism OR humanitarianism OR prosocial behavior OR prosocial behavior OR social behavior OR compassion OR help* OR care OR caring OR empath* OR shar* OR donat* OR comfort*)

Table S2. The testing tools.

**Author (Year)**	**Testing Tool**	
Pintérová et al.（2015） [33]	Social Measurement Rating Questionnaire (SORAD)	Social Measurement Rating Questionnaire (SORAD) includes Influence index, popularity index, tendency index
Metwaly（2015） [34]	The Social Skills scale	The Social Skills scale includes 34 items on 3 subscales: Social Cooperation 、Social Interaction、Social Independence
Sukys et al. (2016) [35]	Prosocial Tendency Scale (PTM-R)	Prosocial Tendency Scale is used to assess the possibility of their participating in prosocial behaviors in various situations. The scale evaluates six kinds of prosocial behaviors: open, anonymous, scary, emotional, submissive and altruistic.
Río et al. (2016) [36]	Interview	"Describe your feelings, thoughts and thoughts about the parkour learning unit you just experienced in physical education class"
Folletoet al. (2016) [37]	children's perception ability and socially accepted image scale.	The scale consists of 24 items, which are divided into four subscales: cognitive and motor ability, social and maternal acceptance.
Pan et al. (2016) [38]	Manual for the ASEBA school-age forms and profifiles（The CBCL）	Calculate the scores of eight scales: anxiety and depression, withdrawal and depression, physical complaints, social problems, thinking problems, attention problems, illegal behaviors and aggressive behaviors.
Gorucu et al. (2016) [39]	Problem Solving Inventory for Children (PSIC)	Included gestures are divided into verbal attack gestures, physical attack gestures, active attack gestures and reactive attack gestures, and an observation form is prepared to record data related to values expressed through behavior.
Ferguson et al. (2016) [40]	A multiple-baseline design across skills	By calculating the percentage of correct performance of athletes' mental skills, the data of each training is combined into one data point, and the possible data points of the whole study are obtained.
Weiss et al. (2016) [41]	Self-Perception Profile for Adolescents (SPPA)；the Behavioral Conduct subscale；	The SPPA consists of five items, and the higher the score, the stronger the perception ability. The Behavioral Conduct subscale includes five items. By tapping young people's views on doing the right thing, avoiding getting into trouble and acting the way they should. The higher the score, the better the perceived behavior.
Phung et al.（2016） [42]	The Lifetime Social Communication Questionnaire	It is a parent reporting tool used by clinicians to quickly screen ASD.
Lang et al.（2016） [43]	Coping Questionnaire for Children and Adolescents； the	ASQ measures the perceived stress of events that teenagers generally experience in their daily lives. The complete ASQ consists of 58 items, representing 10 subscales.

	Adolescent Stress Questionnaire (ASQ)	
Bakır et al.（2017） [44]	Psychological Well-being Scale (WEMWBS) and Enthusiasm Scale	The WEMWBS includes 14 positive items, and high scores indicate a high degree of mental health. The Enthusiasm scale consists of eight items, one of which is graded reversely.
Ruiz-Ariza et al.（2017） [45]	The CREA test、TEIQue-SF	This test uses the ability to explain problems to measure creativity. The test evaluates four factors: well-being, self-control, emotionality and socialization.
Lee et al. (2017) [30]	Interview	Interview
Malinauskas et al. (2017) [46]	Modified Social Responsibility Questionnaire	The questionnaire consists of 14 aspects that state and measure social responsibility. The questionnaire consists of a series of items, reflecting two aspects of social responsibility: respect (six statements) and care and help (eight items).
Parket al. (2017) [47]	Aggression scale Sociality scale Stress scale	Aggressiveness Scale measures the intention or motivation of certain behaviors and thoughts. Social scale covers the intention or motivation of certain behaviors. Stress scale measurement can be defined as the intention or motivation of some thoughts and behaviors of stress factors.
Messler et al. (2018) [48]	the hyperkinetic disorder questionnaire (SBB-HKS) and the KINDL-R questionnaires mental health	The Questionnaire is composed of six subscales (physical health, mental health, self-esteem, friends, family and daily life/school). The higher the total score, the better the health-related quality of life.
Gulati et al. (2019) [49]	Indian Adaptive Child Self-esteem Scale (SEIC)	The SEIC analyzes the simulated evaluation of teachers' participation in children's academic performance, peer behavior, teacher behavior, punctuality, yoga practice and extracurricular activities.
Ryuh et al.（2019） [50]	The Withdrawn Behavior Checklist (WBC) and Social Distance Scale (SDS)	The WBC is used to evaluate the behavior of ID children, and it can be divided into three categories: withdrawal behavior related to friendship, withdrawal behavior related to communication and withdrawal behavior related to self-esteem. Higher scores indicate a higher level of social distance.
Cai et al. (2020) [51]	Social Response Scale (SRS-2), Children Autism Rating Scale (CARS)	SRS-2 is a 65-item scale, which measures the symptoms of autism spectrum disorder.
Cai et al. (2020) [52]	Social Response Scale (SRS-2), Children Autism Rating Scale (CARS)	SRS-2 is a 65-item scale, which measures the symptoms of autism spectrum disorder.
Zwinkels et al. (2020) [53]	Children's Self-perception Scale (SPPC)	It is used to solve a total of six areas: five specific areas of self-perception (i.e., academic ability, social acceptance, physical ability, physical appearance, behavior transmission) and the overall sense of self-worth.
Ringenbach et al.（2020） [54]	Vineland Adaptive Behavior Scale (VABS) II	It is used to assess personal daily living skills, social coping skills, gross exercise, internalized maladjustment and externalized maladjustment.
Condello et al. (2021) [55]	Evaluation scale	MASCS consists of two prosocial subscales (cooperation and empathy) and two antisocial subscales (impatience and destructiveness).
Perić et al. (2021) [56]	the Full Scale Intelligence Quotient (FSIQ)	Use four comprehensive scores (language comprehension, perceptual reasoning, working memory and processing speed) to generate an assessment of the overall intellectual ability.
Jalilinasab et al.（2021） [57]	Matson Evaluation of Social Skills	A rating scale to measure children's social skills.
